# Molecular and Clinical Features of EGFR-TKI-Associated Lung Injury

**DOI:** 10.3390/ijms22020792

**Published:** 2021-01-14

**Authors:** Tohru Ohmori, Toshimitsu Yamaoka, Koichi Ando, Sojiro Kusumoto, Yasunari Kishino, Ryou Manabe, Hironori Sagara

**Affiliations:** 1 Department of Medicine, Division of Respiratory Medicine and Allergology, Showa University School of Medicine, 1-5-8 Hatanodai, Shinagawa-ku, Tokyo 142-8666, Japan; ohmorit@med.showa-u.ac.jp (T.O.); koichi-a@med.showa-u.ac.jp (K.A.); k-sojiro@med.showa-u.ac.jp (S.K.); ookiyookiy@med.showa-u.ac.jp (Y.K.); r.manabe@med.showa-u.ac.jp (R.M.); sagarah@med.showa-u.ac.jp (H.S.); 2 Advanced Cancer Translational Research Institute, Showa University, 1-5-8 Hatanodai, Shinagawa-ku, Tokyo 142-8555, Japan

**Keywords:** EGFR-TKIs, lung injury, inflammation, TNF

## Abstract

The tyrosine kinase activity of epidermal growth factor receptors (EGFRs) plays critical roles in cell proliferation, regeneration, tumorigenesis, and anticancer resistance. Non-small-cell lung cancer patients who responded to EGFR-tyrosine kinase inhibitors (EGFR-TKIs) and obtained survival benefits had somatic EGFR mutations. EGFR-TKI-related adverse events (AEs) are usually tolerable and manageable, although serious AEs, including lung injury (specifically, interstitial lung disease (ILD), causing 58% of EGFR-TKI treatment-related deaths), occur infrequently. The etiopathogenesis of EGFR-TKI-induced ILD remains unknown. Risk factors, such as tobacco exposure, pre-existing lung fibrosis, chronic obstructive pulmonary disease, and poor performance status, indicate that lung inflammatory circumstances may worsen with EGFR-TKI treatment because of impaired epithelial healing of lung injuries. There is limited evidence from preclinical and clinical studies of the mechanisms underlying EGFR-TKI-induced ILD in the available literature. Herein, we evaluated the relationship between EGFR-TKIs and AEs, especially ILD. Recent reports on mechanisms inducing lung injury or resistance in cytokine-rich circumstances were reviewed. We discussed the relevance of cytotoxic agents or immunotherapeutic agents in combination with EGFR-TKIs as a potential mechanism of EGFR-TKI-related lung injury and reviewed recent developments in diagnostics and therapeutics that facilitate recovery from lung injury or overcoming resistance to anti-EGFR treatment.

## 1. Introduction

Lung cancer is the leading cause of death worldwide [1]. Immunotherapy and targeted therapy, in selected populations, has achieved major improvements in the prognosis of patients with advanced non-small cell lung cancer (NSCLC) [2]. Epidermal growth factor receptor (EGFR) mutations, which are detected in up to 50% of patients with lung adenocarcinoma, are recognized as the major actionable target in Asians [3]. Three generations of EGFR-tyrosine kinase inhibitors (TKIs) have been developed and approved for the treatment of advanced NSCLC in patients carrying EGFR-activating mutations. These EGFR-TKIs, which include first-generation (gefitinib and erlotinib), second-generation (afatinib and dacomitinib), and third-generation (osimertinib) agents, have induced improved outcomes, including efficacy and safety, overall when compared with standard chemotherapy [4].

With EGFR-TKI treatment, NSCLC patients who have EGFR-activating mutations can achieve longer progression-free survival (PFS) and a higher quality of life [5,6,7]. The EGFR-TKIs are generally well tolerated because of a favorable toxicity profile compared to chemotherapeutic agents. EGFR plays an essential role in epithelial maintenance and, therefore, EGFR-TKIs might impair epithelial cell growth and migration and alter cytokine expression, leading to the recruitment of inflammatory cells and consequent tissue injury. For example, diarrhea, acneiform skin rash, and paronychia are the commonest side effects of EGFR-TKIs. Mucositis, stomatitis, corneal erosion, and epistaxis are less common, but are clinically important. These side effects are associated with the effect of wild-type-EGFR inhibition. Hepatic and pulmonary toxicity has been reported to be an EGFR-TKI-associated fatal event [8,9]. Severe hepatic dysfunction can be managed by switching to another EGFR-TKI, whereas lung injury (also known as interstitial lung disease (ILD)) often leads to the discontinuation of EGFR-TKI treatment in the affected patients [10]. ILD is a rare but serious complication of EGFR-TKI because one-third of the patients with EGFR-TKI-associated lung injury die, even after receiving intensive supportive care, including supplemental oxygen, empirical antibiotics, systemic corticosteroids, and mechanical ventilation. Therefore, lung injury is the most life-threatening adverse event (AE) related to EGFR-TKI treatment. However, the mechanisms mediating the association between EGFR-TKI treatment and lung injury is not well known. The NSCLC patients with EGFR-TKI-induced lung injury had characteristic clinical profiles of risk factors, including smoking history, male sex, and pre-existing lung fibrosis. On the basis of these patient profiles, it is possible to speculate that lung injury would be worsened by EGFR-TKI treatment in patients with chronic inflammation.

This review focused on the relationship between EGFR-TKIs and AEs, especially lung injury. We reviewed recent reports concerning the mechanisms that induce lung injury or resistance in cytokine-rich circumstances. We discussed the relevance of cytotoxic agents or immunotherapeutic agents in combination with EGFR-TKIs as a potential pathogenic mechanism of EGFR-TKI-related lung injury. Furthermore, we reviewed recent developments in diagnostics and therapeutics that may help to prevent lung injury or to overcome resistance to anti-EGFR treatment.

## 2. Development of EGFR-TKIs and Their AEs

### 2.1. Development of EGFR-TKIs in Clinical Studies

Targeted drug treatment directed at oncogenes has garnered dramatic clinical benefits and has ushered in an era of “targeted therapy”. Similar efforts are underway for cancers with the identification of several potentially promising targetable molecular drivers. Since the discovery of EGFR-activating mutations in NSCLC patients and the elucidation of their response to EGFR-specific TKIs, additional molecular-specific cohorts of NSCLC have been discovered, with the rapid development of targeted drugs specific to each respective abnormality [11]. Treatment with an EGFR-TKI is the current standard of care for patients with locally advanced or metastatic NSCLC who have active mutations.

Currently, several EGFR-TKIs are available for NSCLC patients with EGFR-activating mutations. First-generation EGFR-TKIs (gefitinib and erlotinib) exhibit excellent efficacy. Second-generation covalent inhibitors of EGFR (afatinib and dacomitinib) demonstrate increased cellular inhibitory potency against the driver variants in EGFR [12]. To counter T790M-dependent resistance, a third-generation covalent EGFR-TKI (osimertinib) has been developed with a high potency toward T790M-containing mutants and selectivity over wild-type EGFR [13]. Section 2.1.1, Section 2.1.2, Section 2.1.3 present the background of the development and the results of clinical trials of each generation of EGFR-TKIs.

#### 2.1.1. First-Generation EGFR-TKIs

The Iressa Pan-Asia Study evaluated gefitinib, compared with carboplatin–paclitaxel, as an initial treatment for NSCLC in East Asian non-smokers or former light smokers with pulmonary adenocarcinoma and found that gefitinib treatment prolonged progression-free survival (PFS), increased the objective response rate, reduced toxic effects, and improved the quality of life [7]. Subsequent analysis revealed gefitinib sensitivity in the patient subgroups with EGFR mutation-positive NSCLC. In the North East Japan (NEJ) 002 and West Japan Thoracic Oncology Group 3405 trials of gefitinib versus chemotherapy in patients with EGFR mutation-positive NSCLC, gefitinib extended the PFS (median PFS, 10.8 months for gefitinib vs. 5.4 months for chemotherapy; hazard ratio (HR), 0.30; *p* < 0.001 and median PFS, 9.2 months for gefitinib vs. 6.3 months for chemotherapy; HR, 0.48; *p* < 0.0001, respectively) [14,15]. 

#### 2.1.2. Second-Generation EGFR-TKIs

A randomized phase IIb trial of gefitinib versus afatinib in patients with NSCLC showed that afatinib extended the PFS (median PFS, 11.0 months for afatinib vs. 10.9 months for gefitinib; HR, 0.73; *p* = 0.017), but failed to extend the overall survival (OS; median OS, 27.9 months for afatinib vs. 24.5 months for gefitinib; HR, 0.86; *p* = 0.025) [16,17]. A randomized phase III trial of gefitinib versus dacomitinib in NSCLC patients showed that dacomitinib extended the PFS and OS (median PFS 14.7 months for dacomitinib vs. 9.2 months for gefitinib; HR, 0.59; *p* < 0.0001 and median OS, 34.1 months for dacomitinib vs. 26.8 months for gefitinib; HR, 0.76; *p* = 0.044, respectively) [18,19]. 

#### 2.1.3. Third-Generation EGFR-TKI

A randomized phase III trial of osimertinib versus gefitinib or erlotinib in NSCLC patients (FLAURA study) revealed that osimertinib extended the PFS (median PFS, 18.9 months for osimertinib vs. 10.2 months for gefitinib or erlotinib; HR, 0.46; *p* < 0.001 and median OS 38.6 months for osimertinib vs. 31.8 months for gefitinib or erlotinib; HR, 0.80; *p* = 0.046, respectively) [20,21]. As compared to first-generation EGFR-TKIs, osimertinib has shown superior efficacy in the central nervous system. Based on these results, osimertinib has been used as first-line therapy in NSCLC patients with EGFR-activating mutations [20]. Given its effectiveness and tolerability, osimertinib is a mainstay in the treatment of EGFR mutation-positive NSCLC.

### 2.2. Adverse Events of EGFR-TKIs and Their Management

Molecular targeted drugs such as EGFR-TKIs were initially considered safe anti-cancer drugs with only minor AEs; however, it is known that targeted molecular therapeutic agents can cause serious AEs, including fatal illness. All generations of EGFR-TKIs have similar side-effect profiles, although the frequency and severity of AEs vary by the respective drugs. Rash, paronychia, and diarrhea were the most common AEs reported with first- and second-generation EGFR-TKIs [22]. These drugs inhibit not only active mutant EGFR but also wild-type EGFR, and normal tissues that express EGFR are impaired by the “target effect”. Osimertinib, with selectivity to active mutant EGFR, has been developed and has been associated with relatively mild AEs [20]. Infrequently, serious AEs, including drug-induced lung injury (mainly ILD), occur with all generations of EGFR-TKIs. In this subsection, we describe the AEs of EGFR-TKIs and their management.

#### 2.2.1. Rash, Paronychia, and Stomatitis

Skin disorders are the commonest EGFR-TKI-associated AE and include rashes, such as acne, dry skin, and paronychia. Rash (all grades) associated with EGFR-TKI use is seen in 61–78%, 78–92.4%, 88%, and 58% of patients treated with gefitinib, erlotinib, afatinib, and osimertinib, respectively (Table 1) [14,16,20,23]. Severe (grade 3 or 4) rash is seen in 1–7%, 7–18.1%, 1%, and 1% of patients treated with gefitinib, erlotinib, afatinib, and osimertinib, respectively (Table 1) [14,16,20,23]. Paronychia (all grades) associated with EGFR-TKIs use is seen in 17–33%, 33%, 56%, and 35% of patients treated with gefitinib, erlotinib, afatinib, and osimertinib, respectively [14,16,20,23]. Severe (grade 3 or 4) paronychia is seen in 1%, 1–4.3%, 2%, and <1% of patients treated with gefitinib, erlotinib, afatinib, and osimertinib, respectively [14,16,20,23]. Stomatitis (all grades) associated with EGFR-TKI use is seen in 20–24%, 20%, 64%, and 29% of patients treated with gefitinib, erlotinib, afatinib, and osimertinib, respectively [14,16,20,24]. Severe (grade 3 or 4) stomatitis is seen in <1%, <1%, 4%, and <2% of patients treated with gefitinib, erlotinib, afatinib, and osimertinib, respectively [14,16,20,24]. The management of skin disorders involves both preemptive interventions and treatment after the symptoms occur. The basic preemptive intervention is moisturizing. The moisture content in the skin would have decreased since the initiation of EGFR-TKI treatment, and moisturizers can alleviate this decrease [23]. The use of products that can dry the skin, such as soaps and alcohol-based or perfumed products, should be avoided, shower time should be limited, and the use of lukewarm rather than hot water should be recommended [25]. If signs or symptoms of a rash appear, active application of corticosteroids at an early stage is required. For acne that is refractory to corticosteroid application, an orally administered tetracycline regimen is recommended. For paronychia, preemptive interventions include keeping nails trimmed and avoiding extreme temperatures, friction, or other injuries; taping of fingertips relieves pain. Stomatitis requires preventive oral care and ascertaining the compatibility of dentures. When stomatitis worsens, topical corticosteroids and azulene and Chinese herbal medicine gargles (Ban Xia Xie Xin Tang) are used together.

#### 2.2.2. Diarrhea

Diarrhea (all grades) associated with EGFR-TKI use is seen in 34–61%, 51–58%, 91%, and 58% of patients treated with gefitinib, erlotinib, afatinib, and osimertinib, respectively (Table 1) [14,16,20,24]. Severe (grade 3 or 4) diarrhea is seen in 1–2.2%, 2–3.3%, 13%, and 2% of patients treated with gefitinib, erlotinib, afatinib, and osimertinib, respectively (Table 1) [14,16,20,24]. For diarrhea, orally administered loperamide 4 mg is recommended in the early stage, and for continued episodes of loose stools, loperamide 2 mg every 2 h is administered. In case of poor control, drug holidays and dose reduction should be considered.

#### 2.2.3. Elevated Liver Transaminases

Elevated liver transaminases (all grades) associated with EGFR-TKI use are seen in 25–55%, 27–38%, 10%, and 9% of patients treated with gefitinib, erlotinib, afatinib, and osimertinib, respectively (Table 1) [14,16,20,24]. Severely (grade 3 or 4) elevated liver transaminases is seen in 8–26%, 3.3–8%, 0%, and 1% of patients treated with gefitinib, erlotinib, afatinib, and osimertinib, respectively (Table 1) [14,16,20,24]. Liver disorders are often improved with dose reduction or transient discontinuation of EGFR-TKIs, and/or concomitant use of hepatoprotective agents. Re-administration of EGFR-TKIs often exacerbates liver dysfunction and, in such cases, patients should be switched to other EGFR-TKIs.

#### 2.2.4. Interstitial Lung Disease

Initially, for gefitinib, which was the first approved EGFR-TKI for NSCLC treatment, the AEs were limited to relatively minor events, such as skin disorders. At a time when PCR analysis was not commonly used as a clinical test for detecting EGFR mutations, gefitinib was routinely used non-selectively in NSCLC patients as a safe treatment with orally available tablets. However, after the widespread use of this drug in clinical practice, it was revealed that fatal ILD may be a serious AE caused by gefitinib. The emergence of gefitinib-associated ILD was marked by a lawsuit over drug-induced suffering and was treated as a social problem in Japan. However, in Western countries, where the response rate of NSCLC to gefitinib was relatively low, this drug was not as widely used as in Asian countries. Therefore, in Western countries, ILD, which has a relatively low incidence, has not received much attention, and no large-scale survey has been conducted. However, ILD has been identified as an AE caused by all generations of EGFR-TKIs, and the frequency of treatment-related death is highest with this AE; therefore, ILD is considered to be a non-negligible AE (Table 1) [14,16,20,24]. As with other drug-induced ILDs, the molecular mechanisms of ILD induced by EGFR-TKIs have not been elucidated so far, and the coping method is limited to the use of empirical corticosteroid therapy. Clinical and observational studies in the real-world setting for evaluating EGFR-TKI-related ILD risk factors have been reported. To date, the listed risk factors include male sex, age ≥ 55 years, Eastern Cooperative Oncology Group (ECOG)-Performance Status (PS) ≥ 2, presence of contralateral lung metastases, history of radiation therapy within 1 year, smoking history, early treatment initiation after diagnosis, pre-existing interstitial pneumonia (IP), chronic obstructive pulmonary disease (COPD), lung infectious disease, coexisting heart disease, and normal lung area < 50% [26,27,28,29]. Among these, pre-existing IP was reported as an independent risk factor, and the incidence of gefitinib-induced ILD was significantly higher in this cohort than in the control group (13.9% vs. 3.8%, *p* = 0.013) [30].

## 3. Preclinical Studies of EGFR-TKI Related Lung Injury

The mechanisms of drug-induced lung injury after EGFR-TKI administration are unknown. As mentioned above, EGFR-TKIs block EGFR phosphorylation, thus preventing the regeneration and proliferation of the injured epithelium. This interruption of damage-repair mechanisms by EGFR activation may result in fatal EGFR-TKI-induced lung injury. The clinical features of lung injury induced by EGFR-TKI were identified as pre-existing pulmonary fibrosis, poor PS, and previous thoracic radiation, which were found to be independent risk factors in Japanese NSCLC patients [31]. Moreover, gefitinib-induced ILD was significantly associated with male sex, a history of tobacco use, and the co-occurrence of IP [30]. From these risk factors, it could be speculated that chronic inflammatory conditions in pulmonary tissues could be related to the occurrence of EGFR-TKI-induced lung injury. Most of the pathogenetic mechanisms by which EGFR-TKI-induced lung injury is unknown, although many challenges have been reported in the preclinical and clinical settings.

Preclinically, a murine model of bleomycin-induced pulmonary fibrosis has been employed for detecting the effect of EGFR-TKIs in pulmonary tissue. Suzuki et al. reported that gefitinib treatment of mice augments bleomycin-induced fibrosis. Following 3 weeks of gefitinib administration, severe fibrosis and collagen deposition were observed in the Institute of Cancer Research (ICR) mice that received both bleomycin and the EGFR-TKI, gefitinib, as compared to mice that received bleomycin and vehicle. The mechanisms underlying the augmentation of fibrosis through the inhibition of EGFR tyrosine kinase were unclear; however, it can be suggested that EGFR-TKIs might stimulate apoptosis of alveolar epithelial cells or inhibit epithelial cell differentiation and pulmonary angiogenesis [32]. In contrast, Ishii et al. reported that the EGFR-TKI gefitinib prevented bleomycin-induced lung fibrosis in a C57BL/6 mouse model. Immunohistochemical staining with a fibroblast-specific marker of the S100A4 antibody showed that gefitinib treatment inhibited the EGFR phosphorylation of fibroblasts and significantly decreased fibroblast proliferation, which was induced by bleomycin [33]. In these two research groups, there were no significant differences between the experimental methods, except for the difference in the mice variants used: ICR and C57BL/6. The reason for the discrepancy between these results is unknown. 

Recently, a study using a bleomycin-induced lung fibrosis model showed that metformin attenuated gefitinib-induced exacerbation through the inhibition of TGF-β signaling [34]. Male Sprague–Dawley rats were used for this experiment. Metformin is used to treat type II diabetes, and reduces glycogenesis through adenosine monophosphate-activated kinase (AMPK) signaling, thereby increasing glucose uptake in muscle cells in patients with diabetes, which leads to a decrease in glucose and insulin levels [35]. The rat lungs exhibited fibrosis following intratracheal injection of bleomycin and exacerbation of lung fibrosis by gefitinib administration for 3 weeks. Interestingly, metformin attenuated gefitinib-induced exacerbation of lung fibrosis and collagen deposition. This effect might be associated with a decrease in metformin-mediated activation of TGF-β signaling. In this context, IL-6, which is produced downstream of TGF-β activation, might contribute to the development of acute lung injury [36]. IL-6-deficient mice showed attenuated inflammatory cell (such as macrophages and neutrophils) accumulation after bleomycin administration compared to wild-type mice [37]. Furthermore, EGFR-TKI treatment increased IL-6 secretion in cancer cells, suggesting aggravation of lung injury [38].

Previously, a naphthalene-induced lung injury model was employed to investigate the effect of EGFR-TKI and gefitinib on airway injury and repair. Naphthalene is an aromatic hydrocarbon with Clara cell-selective cytotoxicity. Clara cells, also known as club cells, represent the major secretory cells of the small-airway epithelium in the lung, and play important protective roles through immune modulation, oxidative stress reduction, and metabolism of xenobiotics [39,40,41]. In this study, C57BL/6J mice were injected intraperitoneally with naphthalene, and gefitinib was orally administered for 7 and 14 days, respectively [42]. Naphthalene alone induced neutrophil infiltration at Day 7 but not on Day 14 in the lung tissue, whereas gefitinib administration after naphthalene treatment worsened neutrophil recruitment and acute lung injury was prolonged even on Day 14. Importantly, the terminal epithelial cells were retrieved with laser capture microdissection and gene expression was analyzed using a microarray. Then, 17 genes were determined to have a more than three-fold increase in the bronchiolar epithelial cells obtained from mice gefitinib/naphthalene at Day 14 compared to that from the naphthalene-alone mice. The genes were associated with neutrophil sequestration, acute inflammation, and airway remodeling, such as S100AB, S100A6, and StefinA3. Therefore, the suppression of EGFR signaling leads to the modulation of the expression of pro-inflammatory molecules in the repairing of airway epithelial cells.

**Figure 1:**
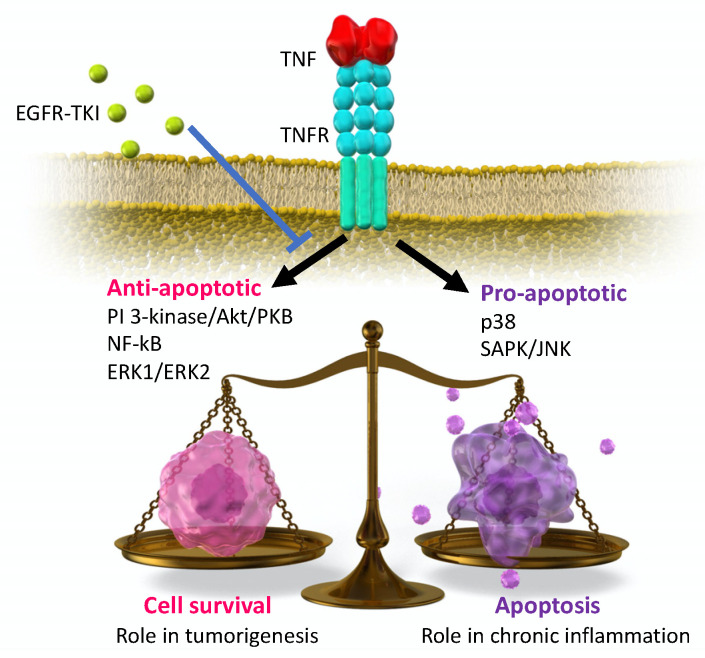
Schematic image of the downstream signaling of tumor necrosis factor (TNF); the balanced regulation of cell survival.

Even with a bleomycin- or naphthalene-induced lung fibrosis/injury model, the best animal model should use EGFR-TKI alone to induce pulmonary injury or fibrosis. Previously, the authors used surfactant protein-C tumor necrosis factor transgenic (SPC-TNF tg) mice to test EGFR-TKI-induced lung injury. In this study, the authors hypothesized that EGFR can be transactivated by many types of extracellular stimuli, such as antagonists of G–protein-coupled receptors (GPCR), cytokines, and cytokine receptors [43]. Tumor necrosis factor (TNF), interleukin (IL)-1β, IL-8, IL-13, and interferon (INF)-γ have been reported to transactivate EGFR in pulmonary epithelial cells through their cytokine receptors [44,45,46]. Therefore, EGFR transactivation through these cytokines might regulate multiple aspects of pulmonary cell homeostasis in response to injury. TNF is a pro-inflammatory cytokine that regulates many biological properties, such as cell survival, apoptosis, proliferation, and migration, in various types of tissues [47]. The downstream signals overlap between EGFR and TNF. TNF, in itself, is not a ligand for EGFR, although TNF stimulates EGFR phosphorylation through EGFR ligands in a dependent or independent manner. Several EGFR ligands are cleaved from the cell surface through the TNF-α-converting enzyme (TACE; also called the disintegrin and metalloproteinase domain-containing protein 17 (ADAM17)) through the process of ectodomain shedding [48]. The TACE-mediated release of EGFR ligands is considered essential for EGFR activation. Cells from the human lung epithelial cell line BEAS-2B showed EGFR transactivation induced by TNF via the cleavage of heparin-binding (HB)-EGF by TACE and were protected from TNF-induced apoptosis [49]. EGFR and HER2 transactivated by TNF promote the survival response of colon epithelial cells through Src-kinase activity [46]. These findings indicate an important relationship between EGFR and TNF signals and epithelial cell survival in the cytokine-rich environment of acute injury response. Moreover, TNF knockout protects against bleomycin-induced lung fibrosis [50]. Therefore, we tested whether EGFR-TKIs enhance the development of lung injury in TNF-overexpressing lung tissues by inhibiting TNF-induced EGFR transactivation. SPC-TNF transgenic mice, a variant generated by Dr. Vasalli in 1995, were used, and transgenic TNF on the SPC promoter was overexpressed only in lung tissue and consequently resulted in alveolitis, alveolar disruption, and subsequent fibrogenesis in lung tissue [51]. Our results demonstrated that gefitinib remarkably enhanced lung inflammation and significant apoptosis induction that were mediated via the p38 MAPK pathway, as observed in SPC-TNF Tg mice. This indicates that EGFR-TKI disrupts the balance of cell survival in TNF-rich conditions through protein kinase B (AKT) and Extracellular Signal-regulated Kinase (ERK)1/2 inhibition as well as the activation of p38 MAP kinase (Figure 1). EGFR-TKIs are administered to patients with NSCLC who had EGFR-activating mutations, and careful attention should be paid to patients with COPD, pneumonitis, pneumonia, and inflammatory cytokine-enriched circumstances when selecting EGFR-TKI treatment. 

Despite the accumulating data and knowledge of EGFR-TKI-related pneumonitis, many issues remain to be solved. The pathophysiology of EGFR-TKI-related pneumonitis has not been elucidated, although a recent observation from a clinical combination trial of a novel immune-checkpoint inhibitor (ICI) and third-generation EGFR-TKI, Osimertinib, raised concerns with regard to lung toxicity [52]. A programmed death-ligand 1 (PD-L1) inhibitor, durvalumab, plus osimertinib in patients with EGFR-mutation positive NSCLC indicated a significantly high rate of lung toxicity, with an incidence of 22% (affecting five out of 23 patients). In addition, patients with advanced NSCLC who were previously treated with nivolumab, a programmed death-1 (PD-1) inhibitor, developed severe pneumonitis during EGFR-TKI administration [53]. These observations may indicate that some suggested mechanisms of EGFR-TKI-related lung injury/fibrosis and further studies are required to determine the risk of combination therapy or sequential use of ICIs and EGFR-TKIs. The underlying mechanisms might involve cell-mediated autoimmune reaction or T–cell-mediated delayed hypersensitivity in ICI, or EGFR-TKI-related lung toxicity [54,55]. Moreover, the disruption of lung remodeling and the impaired response to injury were limited by EGFR-TKIs. These possible mechanisms are complicated by the modification of various host and environmental factors.

## 4. Inflammatory Cytokines on EGFR-TKI Resistance

### 4.1. Expression of Inflammatory Cytokines on EGFR-TKI Resistance

Previous studies have evaluated the association between inflammatory cytokines and EGFR-TKI resistance. For example, a head and neck cancer study reported that neutralization of IL-1M overcame erlotinib resistance in vivo, indicating that blocking IL-1 and downstream signaling could block the development of erlotinib resistance [56]. Moreover, experimental studies in colorectal cancer have found that elevated production of IL-1A, IL-1B, and IL-8, some of the inflammatory cytokines, is associated with increased resistance to cetuximab [57]. Therefore, evaluating the expression levels of these inflammatory cytokines in tumor specimens prior to treatment initiation may help clinicians predict the therapeutic efficacy.

Another study reported that IL-22 positively correlated with acquired resistance to EGFR-TKI in NSCLC patients with EGFR mutations [58]. Moreover, IL-22 has been shown to induce gefitinib resistance both in vitro and in vivo, indicating that IL-22 induction within the tumor microenvironment complicates the acquired EGFR-TKI-resistance in NSCLC [58].

A study of PC-9, a gefitinib-sensitive NSCLC cell line, investigated whether IL-8 was associated with resistance to gefitinib and found that, in addition to IL-8, an IL-8-specific receptor, C-X-C motif chemokine receptor 1 (CXCR1) was significantly upregulated in this cell line. Together, these results suggest that IL-8-CXCR1/2 signaling is associated with gefitinib resistance [59], thus providing a possible mechanism whereby high plasma IL-8 levels mediate PFS in EGFR TKI-treated patients with EGFR mutation-positive lung adenocarcinoma.

Results of previous studies have shown that the clinical outcome of EGFR-TKI treatment is related to the levels of inflammatory cytokines, including plasma IL-8, IL-10, and regulated upon activation, normal T-cell expressed and secreted (RANTES), at the time of diagnosis [60].

We previously examined the relationship between acquired resistance to EGFR-TKI and TNF sensitivity in NSCLC cell lines and found that, with regard to the EGFR-sensitive mutation, a gefitinib-resistant cell line was ~67 times more sensitive to TNF-α than the gefitinib-sensitive cell line. Strikingly, cell lines that regained their resistance to gefitinib had a sensitivity to TNF-α similar to that of the gefitinib-sensitive cell line, suggesting that the sensitivity to TNF-α correlated with gefitinib resistance [61]. In addition, a recent study reported that inhibiting TNF signaling in NSCLC cell lines with EGFR-sensitive mutations increased their sensitivity to EGFR-TKI [62]. 

The results of these studies indicate that a clear relationship exists between inflammatory cytokines and resistance to EGFR-TKI. Thus, understanding the mechanisms that underpin this relationship may help overcome EGFR-TKI resistance. 

### 4.2. Possible EGFR-TKI Resistance Mechanisms in Inflammation

Previous studies have examined the possible mechanisms underlying EGFR-TKI resistance in an inflamed state. For example, studies have shown that Akt phosphorylation is elevated in cells that overexpress IL-8 compared to those with the control condition. The phosphoinositide 3-kinase (PI3K)/AKT pathway has been reported as one of the pivotal downstream effectors of IL-8 signaling and is known to cause tumor progression [63]. Moreover, tumor development may occur via IL-8-initiated activation of CXCR1/2 signaling, leading to activation of NF-κB, which in turn promotes further tumor development [59]. This signaling cascade is associated with IL-8 overexpression-induced EGFR-TKI resistance.

Another study found that IL-22 exposure increased the expression levels of phosphorylated-AKT, EGFR, and ERK in the tumor microenvironment after gefitinib treatment versus the control group [58].

**Figure 2:**
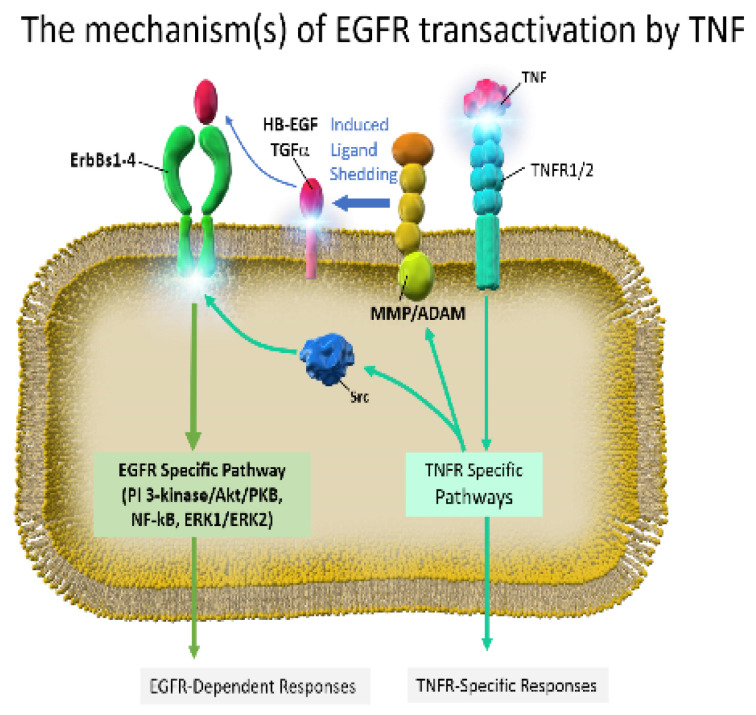
TNF-induced anti-apoptotic cell signaling via tumor necrosis factor receptor (TNFR) to EGFR crosstalk signaling.

We previously examined the association between increased sensitivity to TNF-α and mechanisms of acquired resistance to gefitinib [61]. Our results demonstrated that PI3K/AKT signaling in a gefitinib-sensitive cell line was attenuated by gefitinib in a concentration-dependent manner, whereas PI3K/AKT signaling was unaffected by gefitinib in a gefitinib-acquired resistant cell line. These results suggest that activation of the PI3/AKT signaling by TNF was primarily mediated by constitutive crosstalk signaling from the tumor necrosis factor receptor (TNFR) to EGFR rather than by the direct stimulation from TNFR to PI3/AKT signaling [61]. Therefore, we believe that the increased sensitivity of gefitinib-acquired resistant strains to TNF-α is caused by decreased TNFR-to-EGFR crosstalk signaling, which in turn may enhance TNF-dependent apoptosis (Figure 2).

The crosstalk signaling from TNFR to EGFR is constitutively activated and plays a central role in mediating TNF-activated anti-apoptotic effects via AKT signaling in NSCLC lines with EGFR-sensitive mutations. This crosstalk between TNFR and EGFR is diminished in cell lines that have acquired resistance to gefitinib. In such cases, cells with acquired resistance to gefitinib are sensitive to TNF and are vulnerable to TNF-induced apoptosis. 

A recent study showed that suppression of TNF/NFκB signaling in EGFR-sensitive cell lines affected the sensitivity of EGFR to inhibition, supporting the results of our previous studies [62].

In summary, multiple reports have provided evidence of the relationship between the activation of inflammatory cytokines and the resistance of cancer cells to EGFR-TKIs. Further exploration of the role of inflammatory cytokines in EGFR-TKI resistance could possibly unravel some of the mechanisms by which EGFR-TKI resistance occurs in cancer cells. Further systemic studies incorporating the assessment of inflammatory cytokines could lead to therapeutic strategies that overcome EGFR-TKI resistance.

## 5. Clinical Studies of EGFR-TKI-Related Lung Injury

Here, we present the results of meta-analysis and clinical trials reported so far with regard to the incidence of ILD when there is: (1) EGFR-TKI monotherapy, (2) a combination of EGFR-TKIs and other chemotherapeutic agents, and (3) a combination of EGFR-TKIs and immune checkpoint inhibitors (ICIs). 

### 5.1. EGFR-TKI Monotherapy

Until now, various EGFR-TKI clinical trials have been conducted, and AEs have been investigated in detail. Meta-analyses that included many clinical studies have reported the occurrence of ILD [14,16,23]. The databases of these reports are partially duplicated, as shown in Table 2; however, when racial differences are not taken into account, the incidence of all grades of ILD with EGFR-TKI monotherapy is generally consistent at 1.1–2.2%. The incidence of grade 3 or higher ILD (severe symptoms or oxygen supplemental cases) is 0.6–1.0% and of grade 5 (mortality) AE is 0.2–0.5%. ILD is the most frequent cause of EGFR-TKI-treatment-related death, accounting for 58% of cases [14]. There are no reports of a significant difference in the incidence of ILD between EGFR-TKIs (gefitinib 1.3–2.2%, erlotinib 0.6–1.5%, afatinib 0.2–0.6%, and osimertinib 3.0%) [14,16,23]. 

With regard to racial differences, the incidence of ILD is especially high in the data from Japan. Suh et al. compared research data from Japan and other countries and found that the ILD incidence in Japanese patients was significantly higher, 4.8% vs. 0.6% (*p* < 0.001) for all grades, 2.5% vs. 0.4% (*p* < 0.001) for grade ≥3, and 1.0% vs. 0.2% for grade 5 AEs (*p* < 0.001) [64]. In contrast, when comparing Asian countries, excluding Japanese participants with non-Asians, no significant difference was observed. The reason for the high incidence of ILD in the Japanese population is being analyzed with regard to proteomic [65] and genetic polymorphisms (NEJ022A; UMIN ID: UMIN000015612), although an underlying cause has not been clarified until now. As mentioned earlier, after the occurrence of gefitinib-induced ILD emerged as a social problem in Japan and was evaluated in many clinical studies in Japan as compared to that in other countries, Japanese oncologists have specifically focused on the AE of ILD, and the high incidence may be partly attributable to the high frequency of CT examinations (the number of Japanese CTs per million people is the highest worldwide [66]. Moreover, the lack of quantitative and qualitative international criteria for IP may be a factor, although the diagnostic guideline for idiopathic pulmonary fibrosis (IPF), which represents a collaborative effort between American Thoracic Society (ATS)/European Respiratory Society (ERS)/ Japanese Respiratory Society (JRS)/Latin America Thoracic Association (ALAT), has been issued in 2018 [67].

### 5.2. Combination of EGFR-TKIs and Chemotherapeutic Agents or Angiogenesis Inhibitors

Clinical trial results have been reported on the incidence of ILD when EGFR-TKIs are used in combination with other chemotherapeutic drugs. Hosomi et al. reported that the incidence of ILD in patients receiving carboplatin (CBDCA) + pemetrexed (PEM) + gefitinib was 6% for all grades and 2.4% for grade ≥3 AEs, and the incidence of AEs with gefitinib monotherapy was 3.5% for all grades and 0.6% for grade ≥3 AEs in Japan (NEJ009) [69]. With the determination of the incidence of IP by PEM monotherapy in NSCLC patients at 2.6% in Japan, the combined use of EGFR-TKI and CBDCA + PEM may increase the incidence of ILD compared to EGFR-TKI monotherapy. In contrast, Noronha et al. similarly compared the AE incidence in CBDCA + PEM + gefitinib-administered patients with those of the gefitinib-alone group and found no significant difference in ILD incidence [70].

The incidence of ILD in erlotinib + bevacizumab [71,72,73] and erlotinib + ramucirumab [74] was investigated in patients receiving combination therapy with angiogenesis inhibitors and EGFR-TKIs. None of the patients showed a significant increase in ILD incidence compared to that with erlotinib monotherapy.

### 5.3. Combination of EGFR-TKIs and ICIs

The combination of ICIs with chemotherapeutic drugs has been shown to be effective. In this context, a phase I clinical trial of the anti-programmed death-ligand-1 antibody, durvalumab, and osimertinib combination was conducted [10,75]. When osimertinib was combined with durvalumab 3 or 10 mg/kg, the incidence of ILD was as high as 22% for all grades and 8.7% for grade ≥3 AEs; however, due to the increasing reports of ILD, the clinical trial (TATTON) was terminated. The trial enrolled a relatively small number of patients; however, we infer that combination therapy with EGFR-TKIs and ICIs may increase the incidence of ILD, and development of the combination therapy regimens should be carefully evaluated (Table 3).

## 6. Diagnosis and Therapeutics of EGFR-TKI-Related Lung Injury

### 6.1. Diagnosis

EGFR-TKI-related lung injury, otherwise known as ILD, induced by EGFR-TKI, is a rare but fatal AE. The diagnosis is based on clinical suspicion during EGFR-TKI treatment, detection of lung parenchymal infiltration on radiological assessment, and diagnostic exclusion of tumor progression, cardiac diseases, or other pulmonary complications, such as infectious pneumonitis [55,76]. 

The clinical symptoms of EGFR-TKI-induced ILD include cough, fever, exertional dyspnea, and other respiratory symptoms. As these are non-specific symptoms, and the symptom onset occurs in a wide range of conditions, it is difficult to distinguish ILD from other respiratory diseases. Furthermore, the findings on physical examination are non-specific, although chest auscultation and consideration of other findings, such as peripheral edema, could facilitate the exclusion of a diagnosis of cardiac pulmonary edema [55,76].

In general, chest roentgenography is the first step in the radiological assessment of pulmonary diseases, including ILD. However, the findings are nonspecific and sometimes almost normal in early-stage disease. High-resolution CT (HRCT) of the chest is used in the second step of a diagnostic workup of ILD [55,76], and the findings provide information about not only the distribution pattern but also the abnormalities of the parenchymal pulmonary pattern, which is possibly correlated with the histologic pattern of ILD [77]. Endo et al. analyzed imaging data and clinical manifestations of cases of acute ILD caused by gefitinib, and proposed four radiological patterns based on chest roentgenogram and HRCT (Table 4) [78]. Furthermore, this report showed the incidence of these four patterns that correspond to ILD patterns that reflect a higher mortality risk [78]. An HRCT of the chest should be obtained before the initiation of EGFR-TKI therapy because pre-existing pulmonary abnormalities, such as lung fibrosis, are associated with an increased risk of ILD [30]. Serial assessment of HRCT during EGFR-TKI treatment is helpful for the early detection of ILD-related changes. However, HRCT assessment is not comprehensive because the findings on HRCT of EGFR-TKI-induced ILD are similar to those of other pulmonary complications, such as *Pneumocystis jirovecii* pneumonitis (PCP), cytomegalovirus, and coronavirus disease 2019 (COVID-19) infection [79]. Additionally, patients receiving EGFR-TKIs could be immunocompromised and have a higher risk of infection because of advanced cancer, which can lead to a wrong diagnosis.

Laboratory tests, including complete blood cell counts and biochemical analysis, have been routinely conducted in patients suspected to be at risk for developing ILD. Krebs von den Lungen-6 (KL-6) is a high-molecular weight glycoprotein that is classified as human MUC1 mucin [80]. Elevation of serum KL-6 levels is significantly correlated with various lung diseases such as lung fibrosis and radiation pneumonitis [80,81]. However, evaluating the absolute level of KL-6 in serum is insufficient to diagnose drug-induced ILD [82]. Kawase et al. demonstrated that the degree of elevation of KL-6 from baseline could predict the disease severity as well as the clinical prognosis [83]. Therefore, it is essential to evaluate serum KL-6 levels before the initiation of EGFR-TKI treatment, and close monitoring is necessary during the therapy.

Bronchoscopy is useful to obtain direct information about lung parenchymal abnormalities. However, the main aim of the examination is to exclude infectious pneumonitis and disease progression, and not to confirm the pattern of ILD [84].

### 6.2. Therapeutics

These are no standard guidelines, and no specific therapies have been established for the management of EGFR-TKI-related ILD. Withdrawal of EGFR-TKI treatment is essential in all cases, and supportive treatments with oxygen supplementation are required in patients with respiratory failure. Corticosteroids have been widely used in patients with ILD to control excessive pulmonary inflammation [55,76]. Treatment combining EGFR-TKIs with corticosteroids was successful among patients with NSCLC who experienced ILD induced by EGFR-TKIs [85]. However, such cases were restricted in mild ILD, had non-diffuse alveolar damage (DAD) patterns, and responded well to corticosteroid therapy. On the other hand, it was reported that cases with EGFR-TKI-induced ILD that did not respond to a moderate dose of corticosteroid could have improved with high-dose corticosteroid therapy [86]. High-dose corticosteroid therapy consisted of 500 mg/day, up to 1 g/day, of intravenous methylprednisolone for 3 days. Thereafter, corticosteroids were reduced to a maintenance dose (0.5 to 1 g/kg/day of oral prednisolone). The daily dose of oral prednisolone was decreased by 5–10 mg/week, depending on the patient’s response.

The cytokine interleukin-6 (IL-6) plays a critical role in the inflammatory process and is implicated in the development of severe acute respiratory syndrome [87]. A preclinical investigation demonstrated that EGFR-TKI administration not only decreased the viability of cancer cells, but also increased IL-6 production from cancer cells [38]. The results indicated that EGFR-TKIs could induce ILD via IL-6 production [38]. Therefore, blocking IL-6 activity should be effective for controlling EGFR-TKI-induced ILD. The recombinant humanized anti-human IL-6 receptor monoclonal antibody tocilizumab is widely used for treating autoimmune diseases such as giant cell arthritis [88]. Currently, tocilizumab can achieve a clinical response in patients with severe respiratory failure caused by severe acute respiratory syndrome coronavirus 2 infection [89,90]. No clinical evidence was established in the area of drug-induced ILD; however, a similar pathogenesis should exist. Thus, tocilizumab may be effective in treating severe cases of EGFR-TKI-induced ILD. 

In addition to the previously approved use of corticosteroids as the sole therapy for IPF, pirfenidone and nintedanib have been approved recently and used clinically for the treatment of IPF. In particular, nintedanib has been shown to prevent the development of acute IPF exacerbations [91]. The indications for these anti-fibrotic drugs are currently limited to IPF, although the preventive effect of these drugs with regard to acute exacerbation, due to surgery and chemotherapy, in lung cancer patients with pre-existing IPF is being investigated [92,93]. Kanayama et al. classified 100 patients with indications for surgery into three groups according to the surgical risk score and conducted a retrospective study on the incidence of IPF as an acute exacerbation in the pirfenidone-administered and control groups [92]. The results showed that the incidence of acute exacerbation was suppressed by pirfenidone in all groups. A randomized controlled study is currently underway to evaluate the effect of nintedanib for preventing acute IPF exacerbation during CBDCA + Nab-paclitaxel combination therapy for NSCLC patients with IPF [94]. Thus far, only case studies have reported the effects of anti-fibrotic drugs on EGFR-TKI-related ILD; however, a clinical study of this side effect of EGFR-TKI-related ILD has been planned and will finally help to develop a safer treatment regimen for EGFR-TKI-based therapy.

## 7. Conclusions

The tyrosine kinase activity of EGFR could be an invaluable target for the development of anticancer agents. EGFR-TKI have been developed for NSCLC patients carrying EGFR-activating mutations, such as 15 bp deletions in EGFR exon 19 or L858R mutation in EGFR exon 21. First-generation EGFR-TKIs (such as gefitinib and erlotinib) have exhibited promising survival benefits for NSCLC patients compared to the survival rates observed before EGFR-TKI approval.

However, acquired EGFR T790M mutations can occur as resistance to these EGFR-TKIs. Second-generation EGFR-TKIs (afatinib and dacomitinib) and third-generation EGFR-TKIs (osimertinib) have been developed to overcome such resistant EGFR mutations. Although the development of EGFR-TKIs and combination therapies with EGFR-TKIs are extensively progressive, resistance to EGFR-TKIs still occurs in NSCLC patients with EGFR-activating mutations. The clinical values of these EGFR-TKIs are yet to be completely understood. Furthermore, aberrant EGFR tyrosine kinase activity remains a promising target in cancer therapy.

The EGFR-TKI-induced lung injury occurred at a low frequency of 1.1–2.2%, but it was caused by all generations of EGFR-TKIs and, furthermore, it was fatal, accounting for 58% of all treatment-related deaths of EGFR-TKIs. The risk factor profiles, such as smoking, coincidence of interstitial pneumonia, and COPD, can indicate the circumstances of chronic inflammation in pulmonary tissues. Therefore, the association between inflammatory cytokines and EGFR-TKIs could be involved in the occurrence or progression of EGFR-TKI-induced ling injury. 

Understanding the role of EGFR in cytokine-rich circumstances, such as inflammation, can provide a promising therapeutic strategy to overcome EGFR-TKI-induced lung injury and resistance to EGFR-TKIs.

## Figures and Tables

**Table 1:** Incidence of class-specific adverse events associated with epidermal growth factor receptor (EGFR)-tyrosine kinase inhibitors (TKIs) for treatment of non-small cell lung cancer (NSCLC).

			Rash		Diarrhea		AST/ALT Elevation		ILD	
Trial	Drugs	n	ALL (%)	≥G3 (%)	ALL (%)	≥G3 (%)	ALL (%)	≥G3 (%)	ALL (%)	≥G3 (%)
NEJ002	Gefitinib	144	71.0	5.3	34.2	0.9	55.2	26.3	5.3	2.6
	Carbo/Pacli ^1^	144	19.2	2.7	6.1	0	32.4	0.9	0	0
WJOG 5108L	Gefitinib	277	74.7	2.2	42.6	2.2	42.2/50.9	6.1/13.0	4.3	0.4
	Erlotinib	276	92.4	18.1	51.1	3.3	34.9/38.2	2.2/3.3	4.0	1.4
LUX Lung7	Afatinib	160	88.0	12.0	91.0	13.0	10.0	0	1.3	0.6
	Gefitinib	159	61.0	1.0	61.0	1.0	25.0	9.0	0	0
FLAURA	Osimertinib	279	58	1	58	2	9/6	1/1	4.0	NA ^3^
	Gef. or Erlo ^2^	277	78	7	57	2	25/27	4/8	2.2	NA ^3^

^1^ Carbo/Pacli: carboplatin/paclitaxel, ^2^ Gef. or Erlo: gefitinib or erlotinib, ^3^ NA: not available.

**Table 2:** Incidence of interstitial lung disease (ILD) on EGFR-TKI treatment in meta-analysis.

Author	Patients	Treatments	ILD Incidence (%)	Japanese ILD Incidence (%)	Reference
Suh et al.	15,713	Gef. ^1^, Erlo. ^2^, Afa. ^3^, Osim. ^4^	1.1	4.8	[64]
Shi et al.	8609	Gef. ^1^, Erlo. ^2^	1.2	3.3	[68]
Takeda et al.	1468	Gef. ^1^, Erlo. ^2^, Afa. ^3^	0.6–2.2	3.8	[22]

^1^ Gef.: gefitinib, ^2^ Erlo.: erlotinib, ^3^ Afa.: afatinib, ^4^ Osim.: osimertinib.

**Table 3:** Incidence of ILD in the combined therapy with EGFR-TKIs.

		ILD		
Trial	Drugs	ALL (%)	≥G3 (%)	Reference
NEJ009	CBDCA ^1^ + PEM ^2^ + Gefitinib	11/170 (6.5%)	4/170 (2.4%)	[69]
	Gefitinib	6/171 (3.5%)	1/171 (0.6%)	
NEJ026	Erlotinib + Bevacizumab	0/112 (0%)	0/121 (0%)	[71]
	Erlotinib	5/114 (4.4%)	0/114 (0%)	
RELAY	Erlotinib + Ramucirumab	3/221 (1.4%)	1/221 (0.5%)	[74]
	Erlotinib	4/225 (1.8%)	2/225 (0.9%)	
TATTON	Osimertinib + Durvalumab	5/23 (22%)	2/23 (8.7%)	[75]

^1^ CBDCA: carboplatin, ^2^ PEM: pemetrexed.

**Table 4:** Four proposed radiological patterns based on chest roentgenogram and high-resolution CT (HRCT) on acute ILD caused by gefitinib.

Pattern	Findings on Roentgenography	Manifestations on CT Scan	Corresponding Pattern of ILD	Incidence (%)	Mortality (%)
A	Diffuse and faint opacity without volume loss	Non-specific area: Ground-glass opacity	NSIP ^1^	47.1	31.0
B	Peripheral consolidation	Multifocal area: Airspace consolidation	OP/BOOP ^2^	13.7	28.6
C	Patchy or diffuse faint, liner opacities	Patchy distribution area: Ground-glass opacity interlobular septal thickening	AEP ^3^	2.0	0.0
D	Diffuse faint opacity or consolidation with volume loss	Extensive bilateral area: Ground-glass opacities; air space consolidation with traction bronchiectasis	AIP ^4^	23.5	75.0
Others	Non-specific	Non-specific	N/A	13.7	45.5

^1^ NSIP: nonspecific interstitial pneumonia, ^2^ OP/BOOP: organizing pneumonia/bronchiolitis obliterans organizing pneumonia, ^3^ AEP: acute eosinophilic pneumonia, ^4^ AIP: acute interstitial pneumonia.

## Data Availability

Not applicable.
